# Supplementary material for: Causal Relationship between Aging and Anorexia Nervosa: A White-Matter-Microstructure-Mediated Mendelian Randomization Analysis
Source: Biomedicines. 2024 Aug 16;12(8):1874. doi: 10.3390/biomedicines12081874 (PMC11351342; doi:10.3390/biomedicines12081874)
Supplement: Supplementary file 1 [file biomedicines-12-01874-s001.zip › Supplementary Figure/Figure S8/Leaveoneout plot of MR analysis for TL to ubm-b-1791.pdf]

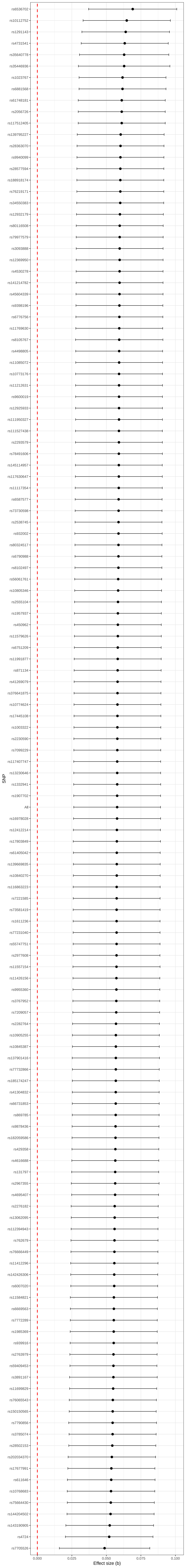
SNP
rs6536702
rs10112752
rs1291143
rs4731541
rs35640778
rs35446936
rs1023767
rs6881568
rs61748181
rs2056726
rs117512405
rs139795227
rs28363070
rs9940099
rs28577594
rs188918174
rs76219171
rs34550383
rs12932179
rs80116508
rs79977579
rs3093888
rs12369950
rs4530278
rs141214782
rs45604339
rs9398196
rs6776756
rs11769630
rs8105767
rs4498805
rs11085072
rs10773176
rs11212631
rs9600019
rs12925933
rs111950327
rs111527438
rs2293579
rs78491606
rs145114957
rs117630647
rs11117354
rs6587577
rs73730598
rs2538745
rs932002
rs80324517
rs6790988
rs8102497
rs56061761
rs10805346
rs2555104
rs1957937
rs450962
rs11579626
rs6751209
rs11991877
rs871134
rs41269079
rs376641875
rs10774624
rs17445108
rs1003322
rs2230590
rs7099229
rs117407747
rs13230646
rs1332941
rs1907702
All
rs16978028
rs12412214
rs17803849
rs61405042
rs139669835
rs10840270
rs116863223
rs7221585
rs73581419
rs1611236
rs77231040
rs55747751
rs2977608
rs11557154
rs11426156
rs9955360
rs3767952
rs7209057
rs2282764
rs10905255
rs10845387
rs137901416
rs77732866
rs185174247
rs41304832
rs66731853
rs869785
rs9878436
rs182059586
rs429358
rs4616688
rs131797
rs2967355
rs4695407
rs2276182
rs13062095
rs112394943
rs762679
rs76666449
rs11412296
rs142426306
rs6007020
rs11584821
rs6669563
rs7772289
rs1985369
rs939916
rs2763979
rs59409453
rs3891167
rs11699829
rs76065543
rs150150565
rs7790856
rs3785074
rs28502153
rs202034370
rs17677991
rs611646
rs10768683
rs75664430
rs144204502
rs143190905
rs4724
rs7705526
0.000
0.025
0.050
0.075
0.100
Effect size (b)
